# Concentration-Dependent Effects of *Artemisia annua* Aqueous Extract on Seed Germination, Seedling Growth, and Physiological Responses of *Solanum rostratum*

**DOI:** 10.3390/plants15193010

**Published:** 2026-09-30

**Authors:** Xiran Li, Xinghua Zhao, Yingmei Ma, Ying Yang, Yuxuan Feng, Yingda Guan

**Affiliations:** 1 College of Desert Control Science and Engineering, Inner Mongolia Agricultural University, Hohhot 010018, China; lxr12342020@163.com (X.L.); mym_youxiang@sina.com (Y.M.); 16604721655@163.com (Y.F.); 15947100076@163.com (Y.G.); 2 Key Laboratory of Desert Ecosystem Conservation and Restoration, State Forestry and Grassland Administration of China, Hohhot 010018, China; 3 Baotou Garden Landscape Research Institute, Baotou 014010, China; y230416@163.com

**Keywords:** *Artemisia annua*, *Solanum rostratum*, seed germination, photosynthesis, oxidative stress, phytotoxicity

## Abstract

This study examined the concentration-dependent effects of an aqueous extract of *Artemisia annua* on seed germination and seedling morphological and physiological traits in the invasive plant *Solanum rostratum*. The extract was also subjected to preliminary chemical profiling using LC–MS/MS. Seed germination was examined in Petri dishes at 50, 100, 150, 200, and 250 g L^−1^, whereas seedling responses were examined in independent plug cells at 100, 150, 200, 250, and 300 g L^−1^. Distilled water served as the control in both experiments. Widely targeted LC–MS/MS profiling of the 300 g L^−1^ extract yielded 9987 putative metabolite annotation entries across ten superclasses, with phenylpropanoids, alkaloids, and terpenoids, constituting the three largest superclasses by number of annotation entries. Seed germination was suppressed at all tested concentrations and completely inhibited at ≥150 g L^−1^. Seedling responses were concentration-dependent: several morphological indices increased at 100–150 g L^−1^ while gas-exchange performance declined, whereas morphological inhibition became increasingly evident at ≥200 g L^−1^. Net photosynthetic rate, stomatal conductance, and transpiration rate declined with increasing extract concentration and were significantly lower under all extract treatments than in the control. Chlorophyll a and chlorophyll b contents initially increased and then decreased, with both peaking at 150 g L^−1^. Carotenoid content increased across the concentration gradient, whereas intercellular CO_2_ concentration exceeded the control only at ≥200 g L^−1^. Electrolyte leakage and the contents of malondialdehyde, H_2_O_2_, and proline generally increased with extract concentration, with the highest values observed at 300 g L^−1^. Catalase activity was significantly lower than that of the control at ≥200 g L^−1^. At high concentrations, morphological inhibition coincided with reduced photosynthetic performance, changes in antioxidant enzyme activities, and increased indicators of oxidative stress and membrane damage. These findings provide laboratory evidence of the phytotoxic potential of *A. annua* aqueous extract but do not establish its efficacy or safety under field conditions.

## 1. Introduction

Alien invasive plants threaten biodiversity, ecosystem functioning, and economic activities, creating an urgent need for effective and environmentally responsible management strategies [1,2]. *Solanum rostratum* Dunal (Solanaceae), which is native to North America, has invaded parts of Asia and Europe, as well as South Africa and Oceania [3]. Its broad environmental tolerance and prolific seed production facilitate its spread. Since its first recorded occurrence in Chaoyang County, Liaoning Province, in the 1980s, *S. rostratum* has been reported from 12 provincial-level regions in China, including Jilin, Inner Mongolia, Beijing, Hebei, and Shanxi [3]. Rapid growth and strong resource-acquisition capacity may confer a competitive advantage over native vegetation. For example, *S. rostratum* has been reported to reduce the growth and biomass accumulation of the native species *Leymus chinensis* and *Agropyron cristatum* [4]. The species also poses risks to human and animal health. It contains toxic glycoalkaloids, including solanine, and ingestion of large quantities by livestock may cause motor dysfunction, diarrhea, vomiting, and enteritis. Its pollen can induce allergic rhinitis in humans, while its spines may injure the skin and mucous membranes of livestock and damage hides [5]. *S. rostratum* serves as a host for the Colorado potato beetle, potentially facilitating the spread of this agricultural pest and associated crop losses [3,6,7]. Its invasion can threaten native vegetation, agricultural and livestock production, and ecosystem functioning [3,6,7].

Current management strategies for *S. rostratum* include physical, chemical, and biological control. Physical control relies primarily on manual or mechanical removal, which requires substantial labor and time. Chemical herbicides can rapidly suppress target plants but may have unintended environmental effects. Biological approaches include the use of natural enemies and pathogenic microorganisms, as well as replacement control through the establishment of competitive plant species [8]. For example, candidate pathogenic fungi have been isolated from naturally diseased *S. rostratum* plants and evaluated for pathogenicity and non-target safety [8]. However, the release of natural enemies requires rigorous assessment of host specificity and potential ecological risks. The effectiveness of replacement control depends on competition for light, water, and nutrients and may vary with community composition and habitat conditions. These limitations have encouraged research into plant-derived bioactive materials as possible complementary approaches to invasive-plant management.

Allelopathy refers to the stimulatory or inhibitory effects of plant-released secondary metabolites on the germination, growth, or development of the producing plant or neighboring plants [9]. Plants release allelochemicals through volatilization, leaching from aboveground tissues, root exudation, and residue decomposition. These compounds may interfere with photosynthesis, reduce root activity, and impair nutrient uptake in recipient plants [10,11,12]. Some allelochemicals have been reported to inhibit glycolysis, the pentose phosphate pathway, and mitochondrial respiration. They may also interfere with electron transport, oxidative phosphorylation, and ATP synthesis, thereby limiting the energy and metabolic substrates required for seed germination and seedling growth [13]. Other allelochemicals have been reported to act on components of the photosystem II electron transport chain, obstruct electron transfer between plastoquinones, or interfere with the biosynthesis of photosynthetic pigments such as carotenoids. Such effects may reduce light capture and photosynthetic efficiency [13]. Certain phenolic, quinone, and flavonoid compounds have been reported to promote the accumulation of reactive oxygen species (ROS). Excessive ROS accumulation has been associated with membrane depolarization, lipid peroxidation, increased membrane permeability, and damage to proteins and DNA [13]. Recipient plants may respond to oxidative stress through changes in antioxidant enzyme activities, including those of superoxide dismutase (SOD), peroxidase (POD), and catalase (CAT). However, when ROS production exceeds the capacity of antioxidant defenses, oxidative cellular damage may occur [13].

*Artemisia annua* L. (Asteraceae) contains diverse secondary metabolites, including terpenoids, flavonoids, coumarins, organic acids, phenolic acids, and artemisinin [14,15]. Some compounds reported in *A. annua* have shown phytotoxic or allelopathic activity in experimental studies [11,12,16]. For example, exposure to artemisinin reduced net photosynthetic rate and relative water content in *Hordeum distichon* seedlings, increased leaf electrolyte leakage, and altered antioxidant enzyme activities [16]. These responses indicate that artemisinin exposure is associated with changes in photosynthetic performance, membrane integrity, and antioxidant responses in recipient plants. Some coumarins have also been reported to inhibit seed germination, root growth, water uptake, respiration, photosynthesis, and cell division and elongation [12]. At the extract level, *Artemisia santolinifolia* extracts inhibited seed germination and seedling growth in *Sinapis arvensis*, *Lolium multiflorum*, and *Parthenium hysterophorus*, while higher extract concentrations reduced chlorophyll content and increased indicators of oxidative stress [17]. These studies indicate that compounds and crude extracts derived from *Artemisia* species can affect the germination, growth, and physiological status of recipient plants. However, these effects depend on the donor species, extract composition, concentration, and recipient plant and therefore require evaluation in specific donor–recipient systems.

The effects of *A. annua* aqueous extract on seed germination and seedling development in *S. rostratum* remain poorly understood. It is also unclear whether changes in seedling morphological traits following exposure to the extract are accompanied by changes in photosynthetic performance, photosynthetic pigment contents, indicators of oxidative stress, membrane integrity, proline content, and antioxidant enzyme activities.

The present study examined the effects of *A. annua* aqueous extract on *S. rostratum*. The specific objectives were to (1) preliminarily characterize the metabolite composition of the 300 g L^−1^ *A. annua* aqueous extract using LC–MS/MS; (2) characterize the concentration-dependent effects of the extract on seed germination and seedling morphological traits within the concentration range tested in each assay; and (3) examine whether changes in seedling morphological traits were accompanied by changes in photosynthetic performance, photosynthetic pigment contents, indicators of oxidative stress, membrane integrity, proline content, and antioxidant enzyme activities.

## 2. Results

### 2.1. LC–MS/MS Characterization of the Artemisia annua Aqueous Extract

After the exclusion of six internal-standard records, widely targeted LC–MS/MS analysis yielded 9987 putative metabolite annotation entries. These entries were assigned to ten superclasses. Phenylpropanoids contained the largest number of entries (2370, 23.73%), followed by alkaloids (2334, 23.37%) and terpenoids (2259, 22.62%). As shown in Table 1, the remaining entries were assigned to lipids (1084, 10.85%), amino acids and derivatives (674, 6.75%), polyketides (510, 5.11%), others (438, 4.39%), carbohydrates (128, 1.28%), organic acids and derivatives (120, 1.20%), and steroids and steroid derivatives (70, 0.70%).

The annotation entries were ranked according to their mean LC–MS/MS peak-area responses across the three analyzed samples. The ten highest-ranked entries are presented in Table 2. Heraclenol ranked first, followed by 10-methyl-2H,3H,4H,7H,10H-pyrano [3,2-c]quinoline-3,5-diol and β-D-glucosylisoeugenol.

The complete annotation dataset also included putative annotations for artemisinin, isoscopoletin, umbelliferone, luteolin-7-glucoside, chlorogenic acid derivatives, artemetin, kaempferide, and eugenol. All metabolite assignments reported in this section were putative and were not confirmed using authentic standards; the corresponding peak areas represent relative instrumental responses rather than metabolite concentrations.

### 2.2. Effects of Artemisia annua Aqueous Extract on Seed Germination

**Figure 1:**
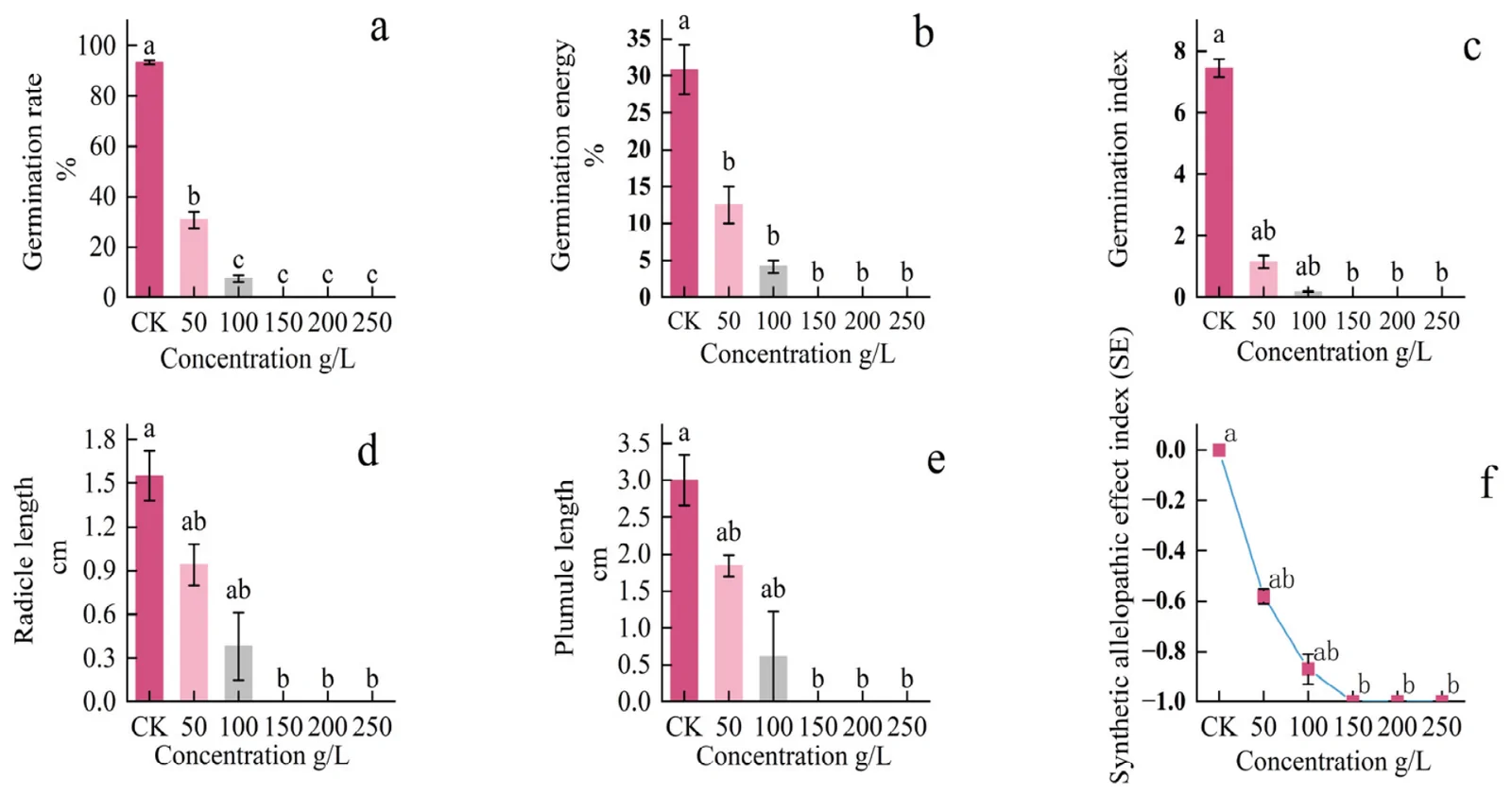
Effects of *Artemisia annua* aqueous extract on seed germination in *Solanum rostratum*. Note: Data are presented as observed means ± SE (*n* = 3 independent Petri dishes per treatment, with 40 seeds per dish). For germination rate (**a**) and germination energy (**b**), different lowercase letters indicate significant differences based on Firth-type mean bias-reduced binomial generalized linear models followed by Holm-adjusted pairwise comparisons (*p* < 0.05). For germination index, radicle length, plumule length, and the synthetic allelopathic effect index (**c**–**f**), treatments with no lowercase letters in common differ significantly (*p* < 0.05), based on rank-based permutation tests with pairwise comparisons adjusted using the maximum absolute difference in mean ranks.

Aqueous extract concentration significantly affected both the germination rate (GR; Wald χ^2^ = 145.608, df = 5, *p* < 0.0001) and germination energy (GE; Wald χ^2^ = 50.786, df = 5, *p* < 0.0001) of *S. rostratum*. Holm-adjusted pairwise comparisons showed that GR was significantly lower under all extract treatments than under the control (CK). GR under the 50 g L^−1^ treatment was significantly higher than that under the 100–250 g L^−1^ treatments, whereas no significant differences were detected between the 100 g L^−1^ treatment and the treatments with no observed germination at 150–250 g L^−1^ (Figure 1a). GE was significantly lower under all extract treatments than under the CK, but no significant differences were detected among the extract treatments after Holm adjustment (Figure 1b). The observed GR decreased from 93.33% in the CK to 30.83% and 7.50% under the 50 and 100 g L^−1^ treatments, respectively. The observed GE decreased from 30.83% in the CK to 12.50% and 4.17% under the 50 and 100 g L^−1^ treatments, respectively. The germination index decreased from 7.45 in the CK to 1.15 and 0.18 under the 50 and 100 g L^−1^ treatments, respectively; only the 150–250 g L^−1^ treatments differed significantly from the CK after adjustment for all pairwise comparisons (Figure 1c). At extract concentrations ≥150 g L^−1^, GR, GE, and the germination index were all zero.

The recorded radicle and plumule lengths decreased with increasing extract concentration (Figure 1d,e). For both variables, only the 150–250 g L^−1^ treatments differed significantly from the CK after adjustment for all pairwise comparisons (*p* < 0.05). Radicle length decreased from 1.55 cm in the CK treatment to 0.94 and 0.38 cm under the 50 and 100 g L^−1^ treatments, respectively. Similarly, plumule length decreased from 3.00 cm in the CK treatment to 1.84 and 0.61 cm under the 50 and 100 g L^−1^ treatments, respectively. At extract concentrations ≥150 g L^−1^, both radicle and plumule lengths were zero, and no seeds germinated.

The synthetic allelopathic effect index was negative at all extract concentrations, indicating an overall inhibitory effect on *S. rostratum* seed germination relative to the CK (Figure 1f). The index values were −0.58 and −0.87 under the 50 and 100 g L^−1^ treatments, respectively; neither differed significantly from the CK after adjustment for all pairwise comparisons (*p* > 0.05). At concentrations of 150, 200, and 250 g L^−1^, the index reached its minimum value of −1.00. The index values under these three treatments differed significantly from the CK (*p* < 0.05), but not from the 50 or 100 g L^−1^ treatments; no significant differences were detected among the three higher-concentration treatments.

### 2.3. Seedling Morphological Responses

**Figure 2:**
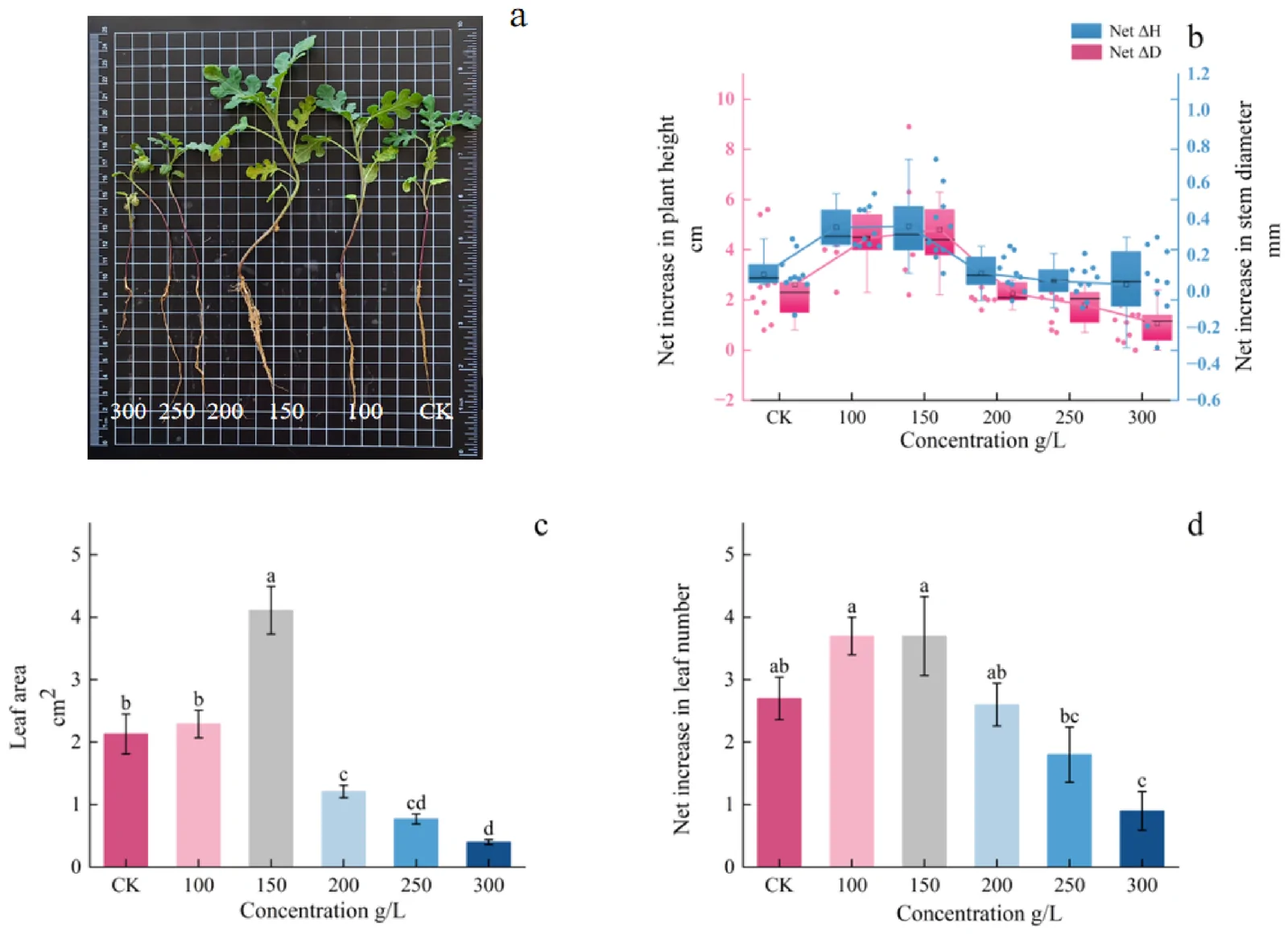
Aboveground morphological responses of *Solanum rostratum* seedlings to different concentrations of *Artemisia annua* aqueous extract. Note: (**a**) representative photographs of seedlings under different treatments; (**b**) net increases in plant height and basal diameter; (**c**) leaf area; (**d**) net increase in leaf number. Different lowercase letters indicate significant differences among treatments at *p* < 0.05. Data are presented as means ± SE (*n* = 10 independent seedlings per treatment for plant height, basal diameter, and leaf number; *n* = 5 independent seedlings per treatment for leaf area).

Several aboveground morphological traits of *Solanum rostratum* seedlings showed biphasic concentration-dependent responses to the *Artemisia annua* aqueous extract, with higher values at lower concentrations and lower values at higher concentrations (Figure 2 and Table 3). The net increases in plant height and basal diameter showed similar response patterns (Figure 2b). The net increase in plant height was 2.61 cm in the control. At 100 and 150 g L^−1^, it increased significantly to 4.46 and 4.80 cm, respectively (*p* < 0.05). As the extract concentration increased further, the net increase in plant height declined to 2.25 and 1.80 cm at 200 and 250 g L^−1^, respectively; however, neither value differed significantly from the control (*p* > 0.05). At 300 g L^−1^, the net increase in plant height decreased to 1.06 cm and was significantly lower than that of the control (*p* < 0.05). The net increase in basal diameter was 0.10 mm in the control and increased significantly to 0.35 and 0.36 mm at 100 and 150 g L^−1^, respectively (*p* < 0.05). With further increases in extract concentration, the net increase in basal diameter declined to 0.10, 0.06, and 0.04 mm at 200, 250, and 300 g L^−1^, respectively, although these values did not differ significantly from the control (*p* > 0.05).

Both leaf area and the net increase in leaf number exhibited biphasic concentration-dependent responses (Figure 2c,d). At both 100 and 150 g L^−1^, the net increase in leaf number was 3.7 leaves, representing a 37.00% increase relative to the control. Thereafter, it declined progressively with increasing extract concentration, reaching 0.90 leaves at 300 g L^−1^, which was 67.00% lower than the control (*p* < 0.05). Leaf area increased at 100 and 150 g L^−1^ and reached a maximum of 4.11 cm^2^ at 150 g L^−1^, representing a significant increase of 93.00% relative to the control (*p* < 0.05). At concentrations of 200, 250, and 300 g L^−1^, leaf area was significantly lower than that of the control, with values of 1.21, 0.77, and 0.40 cm^2^, respectively (*p* < 0.05).

The net increase in leaf thickness also exhibited a biphasic response to increasing extract concentration (Table 3). The net increase was 0.05 mm in the CK treatment. At 100 and 150 g L^−1^, the net increase in leaf thickness was greater than that in the CK, and the maximum value of 0.10 mm was recorded at 150 g L^−1^; only the value at 150 g L^−1^ was significantly higher than the CK value (*p* < 0.05). At 200, 250, and 300 g L^−1^, the net increase in leaf thickness decreased to 0.05, 0.05, and 0.03 mm, respectively, with no significant differences from the CK treatment (*p* > 0.05).

Leaf length and width reached their maximum values of 2.97 and 2.21 cm, respectively, under the 150 g L^−1^ treatment and were significantly greater than those of the CK treatment (*p* < 0.05). At extract concentrations ≥200 g L^−1^, both leaf length and width decreased progressively with increasing concentration. The lowest values were recorded at 300 g L^−1^, where leaf length and width were 0.83 and 0.68 cm, respectively.

**Figure 3:**
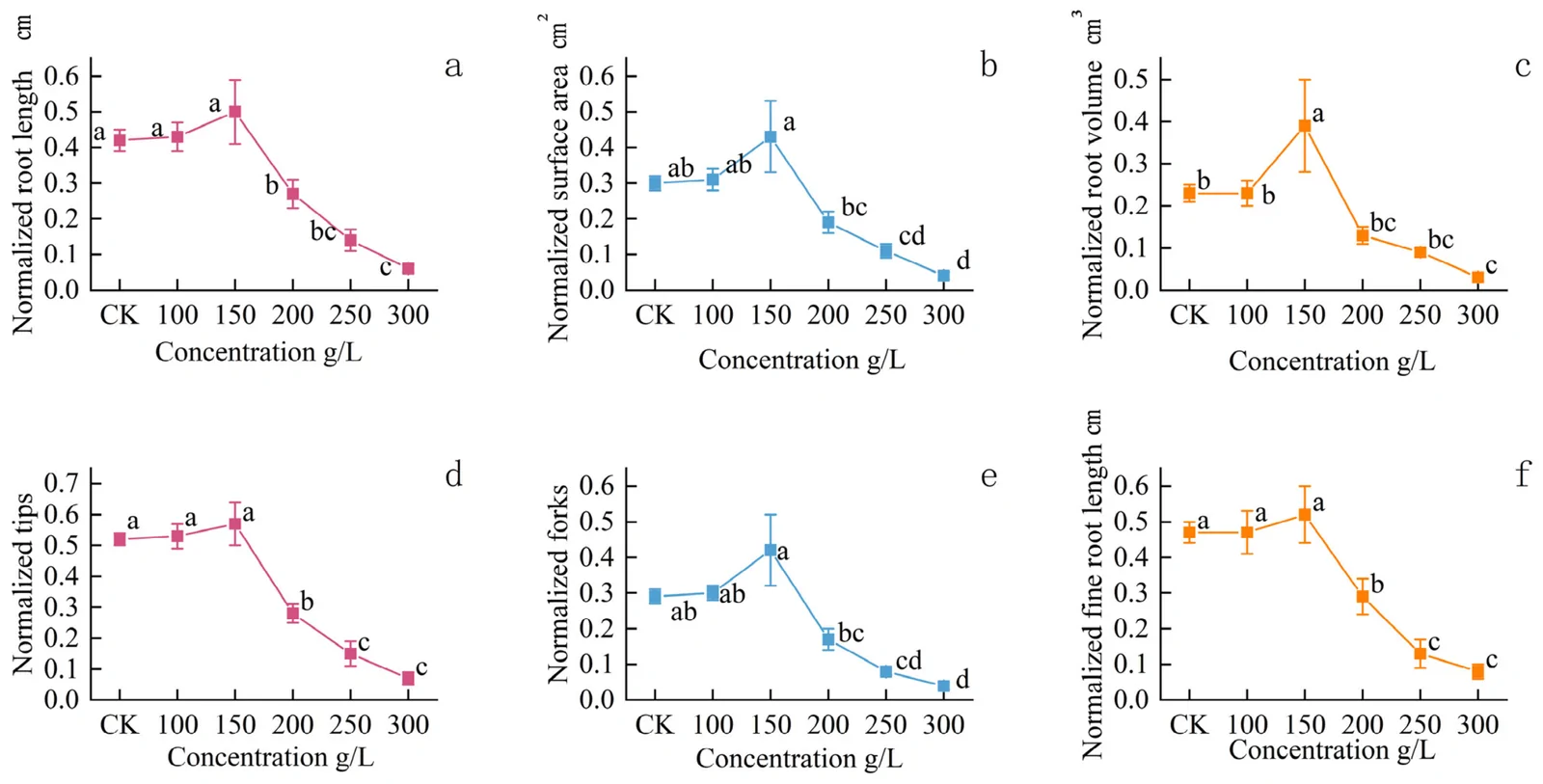
Effects of *Artemisia annua* aqueous extract on root morphological traits of *Solanum rostratum* seedlings. Note: (**a**) normalized root length; (**b**) normalized root surface area; (**c**) normalized root volume; (**d**) normalized number of root tips; (**e**) normalized number of root forks; (**f**) normalized fine root length. Different lowercase letters indicate significant differences among treatments at *p* < 0.05. Data are presented as means ± SE (*n* = 7 independent seedlings per treatment).

Total root length, root surface area, and root volume exhibited biphasic responses to increasing concentrations of *A. annua* aqueous extract (Figure 3a–c). Total root length was numerically higher under the 100 and 150 g L^−1^ treatments than under the CK treatment, although the differences were not significant (*p* > 0.05; Figure 3a). At concentrations of 200–300 g L^−1^, however, total root length was significantly lower than that in the CK (*p* < 0.05). Total root length was 22.21, 13.20, and 8.26 cm under the 200, 250, and 300 g L^−1^ treatments, respectively.

Root surface area was also numerically higher under the 100 and 150 g L^−1^ treatments, with increases of 0.10 and 2.30 cm^2^ relative to the CK treatment, respectively; however, these differences were not significant (*p* > 0.05; Figure 3b). At 200 g L^−1^, root surface area decreased to 3.90 cm^2^, which was 2.02 cm^2^ lower than that of the CK treatment, although the difference was not significant (*p* > 0.05). At 250 and 300 g L^−1^, root surface area was significantly lower than that in the CK, with values of 2.44 and 1.23 cm^2^, respectively (*p* < 0.05).

Root volume was 0.09 cm^3^ in the CK treatment (Figure 3c). Root volume was higher under the 100 and 150 g L^−1^ treatments and reached a maximum of 0.15 cm^3^ at 150 g L^−1^; this value was significantly higher than that of the CK treatment (*p* < 0.05). Root volume subsequently decreased to 0.05 and 0.04 cm^3^ under the 200 and 250 g L^−1^ treatments, respectively. Although these values were numerically lower than those of the CK treatment, the differences were not significant (*p* > 0.05). At 300 g L^−1^, root volume decreased significantly to 0.02 cm^3^ relative to the CK (*p* < 0.05).

The number of root tips, the number of root forks, and the length of roots with diameters ≤0.5 mm showed similar biphasic, concentration-dependent responses to the aqueous extract (Figure 3d–f). The number of root tips under the 100 and 150 g L^−1^ treatments was numerically higher than that under the CK treatment, although the differences were not significant (*p* > 0.05; Figure 3d). At concentrations of 200, 250, and 300 g L^−1^, the number of root tips was significantly lower than that in the CK, with values of 76.29, 43.43, and 23.43, respectively (*p* < 0.05).

Similarly, the number of root forks under the 100 and 150 g L^−1^ treatments was numerically higher than that under the CK treatment, but the differences were not significant (*p* > 0.05; Figure 3e). At 200 g L^−1^, the number of root forks decreased to 176.43, although it did not differ significantly from that of the CK treatment (*p* > 0.05). At 250 and 300 g L^−1^, the number of root forks was significantly lower than that in the CK, with values of 80.00 and 40.71, respectively (*p* < 0.05).

The length of roots with diameters ≤0.5 mm was 22.41 cm in the CK treatment and increased slightly to 22.51 and 24.83 cm under the 100 and 150 g L^−1^ treatments, respectively; however, neither value differed significantly from that of the CK treatment (*p* > 0.05; Figure 3f). At concentrations of 200, 250, and 300 g L^−1^, the length of these roots was significantly lower than that in the CK, with values of 14.79, 8.21, and 6.19 cm, respectively (*p* < 0.05).

### 2.4. Effects of Artemisia annua Aqueous Extract on the Photosynthetic Characteristics of Solanum rostratum

**Figure 4:**
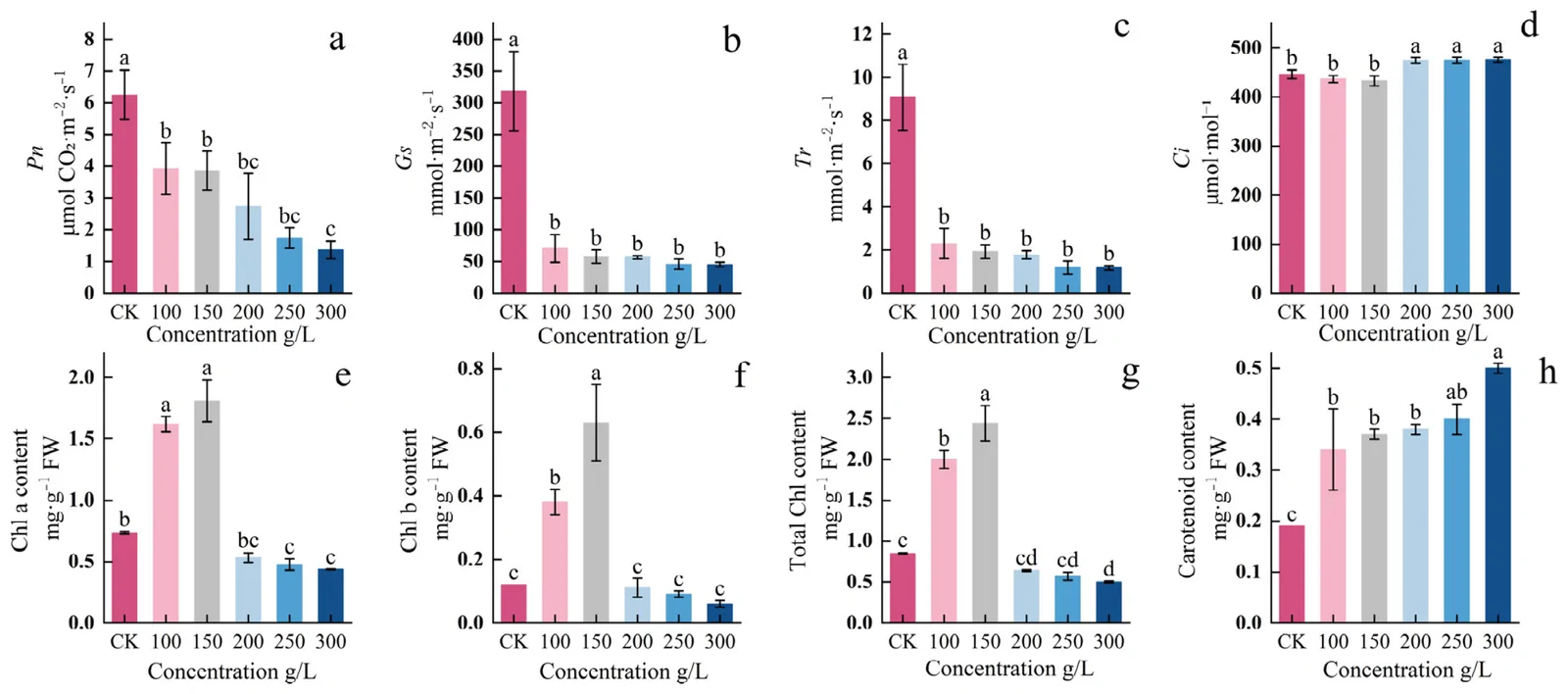
Effects of *Artemisia annua* aqueous extract on the gas-exchange parameters and photosynthetic pigment contents of *Solanum rostratum* seedlings. Note: (**a**) net photosynthetic rate; (**b**) stomatal conductance; (**c**) transpiration rate; (**d**) intercellular CO_2_ concentration; (**e**) chlorophyll a content; (**f**) chlorophyll b content; (**g**) total chlorophyll content; (**h**) carotenoid content. Different lowercase letters indicate significant differences among treatments at *p* < 0.05. Data are presented as means ± SE. For the gas-exchange parameters (**a**–**d**), *n* = 5 independent seedlings per treatment; for the photosynthetic pigment contents (**e**–**h**), *n* = 3 independent seedlings per treatment.

The gas-exchange parameters of *S. rostratum* seedlings varied with the concentration of the *A. annua* aqueous extract (Figure 4). Net photosynthetic rate (*P_n_*) decreased with increasing extract concentration and was significantly lower under all extract treatments than under the CK treatment (*p* < 0.05; Figure 4a). *P_n_* was 6.24 μmol·m^−2^·s^−1^ in the CK treatment and decreased to 3.92 and 3.86 μmol·m^−2^·s^−1^ under the 100 and 150 g L^−1^ treatments, respectively. No significant difference was detected between these two treatments (*p* > 0.05), although both values were significantly lower than the CK value (*p* < 0.05). As the extract concentration increased to 200, 250, and 300 g L^−1^, *P_n_* decreased further to 2.73, 1.73, and 1.36 μmol·m^−2^·s^−1^, respectively. Stomatal conductance (*g_s_*) was 318.07 mmol·m^−2^·s^−1^ in the CK treatment (Figure 4b). Under the 100, 150, 200, 250, and 300 g L^−1^ treatments, *g_s_* decreased to 70.58, 57.77, 56.41, 45.41, and 44.97 mmol·m^−2^·s^−1^, respectively. The *g_s_* values were significantly lower under all extract treatments than under the CK treatment (*p* < 0.05), whereas no significant differences were detected among the extract treatments (*p* > 0.05). A similar response was observed for transpiration rate (*T_r_*; Figure 4c). *T_r_* was 9.06 mmol·m^−2^·s^−1^ in the CK treatment and decreased to 2.30, 1.93, 1.78, 1.19, and 1.16 mmol·m^−2^·s^−1^ under the 100, 150, 200, 250, and 300 g L^−1^ treatments, respectively. *T_r_* was significantly lower under all extract treatments than under the CK treatment (*p* < 0.05). The response of intercellular CO_2_ concentration (*C_i_*) differed from the responses of the other gas-exchange parameters (Figure 4d). *C_i_* was 446.34 μmol·mol^−1^ in the CK treatment. Under the 100 and 150 g L^−1^ treatments, *C_i_* decreased slightly to 437.03 and 433.09 μmol·mol^−1^, respectively, but neither value differed significantly from the CK value (*p* > 0.05). At extract concentrations of 200, 250, and 300 g L^−1^, *C_i_* increased to 474.40, 474.57, and 475.55 μmol·mol^−1^, respectively. No significant differences were detected among these three treatments (*p* > 0.05); however, their *C_i_* values were significantly higher than those under the CK, 100 g L^−1^, and 150 g L^−1^ treatments (*p* < 0.05).

At the lower extract concentrations, seedling morphological and gas-exchange responses were not fully synchronized. Although the net increases in plant height and basal diameter were significantly greater under the 100 and 150 g L^−1^ treatments than under the CK, *P_n_*, *g_s_*, and *T_r_* were already significantly lower than the corresponding CK values (*p* < 0.05).

Leaf photosynthetic pigment contents also varied with extract concentration (Figure 4e–h). Chlorophyll a content was 0.73 mg·g^−1^ in the CK treatment (Figure 4e) and increased significantly to 1.62 and 1.81 mg·g^−1^ under the 100 and 150 g L^−1^ treatments, respectively (*p* < 0.05). Chlorophyll a content subsequently decreased with increasing extract concentration. At 200 g L^−1^, chlorophyll a content decreased to 0.53 mg·g^−1^ but did not differ significantly from the CK value (*p* > 0.05). At 250 and 300 g L^−1^, chlorophyll a content decreased to 0.48 and 0.44 mg·g^−1^, respectively, and was significantly lower than the CK value (*p* < 0.05).

Chlorophyll b content was 0.12 mg·g^−1^ in the CK treatment (Figure 4f). Chlorophyll b content increased significantly to 0.38 and 0.63 mg·g^−1^ under the 100 and 150 g L^−1^ treatments, respectively (*p* < 0.05). At concentrations of 200, 250, and 300 g L^−1^, chlorophyll b content decreased to 0.11, 0.09, and 0.06 mg·g^−1^, respectively. Although these values were lower than the CK value, the differences were not significant (*p* > 0.05).

Total chlorophyll content exhibited a biphasic concentration-dependent response (Figure 4g). It increased from 0.85 mg·g^−1^ in the CK treatment to 2.00 and 2.44 mg·g^−1^ under the 100 and 150 g L^−1^ treatments, respectively, and both values were significantly higher than the CK value (*p* < 0.05). Total chlorophyll content subsequently decreased to 0.64 and 0.57 mg·g^−1^ under the 200 and 250 g L^−1^ treatments, respectively; however, neither value differed significantly from the CK value (*p* > 0.05). At 300 g L^−1^, total chlorophyll content was significantly lower than the CK value, decreasing to 0.50 mg·g^−1^ (*p* < 0.05).

In contrast to the chlorophyll response, carotenoid content increased progressively with increasing extract concentration (Figure 4h). Carotenoid content was 0.19 mg·g^−1^ in the CK treatment and increased to 0.34, 0.37, 0.38, 0.40, and 0.50 mg·g^−1^ under the 100, 150, 200, 250, and 300 g L^−1^ treatments, respectively. Carotenoid content was significantly higher under all extract treatments than under the CK treatment (*p* < 0.05).

### 2.5. Effects of Artemisia annua Aqueous Extract on Stress-Related Physiological Responses in Solanum rostratum

**Figure 5:**
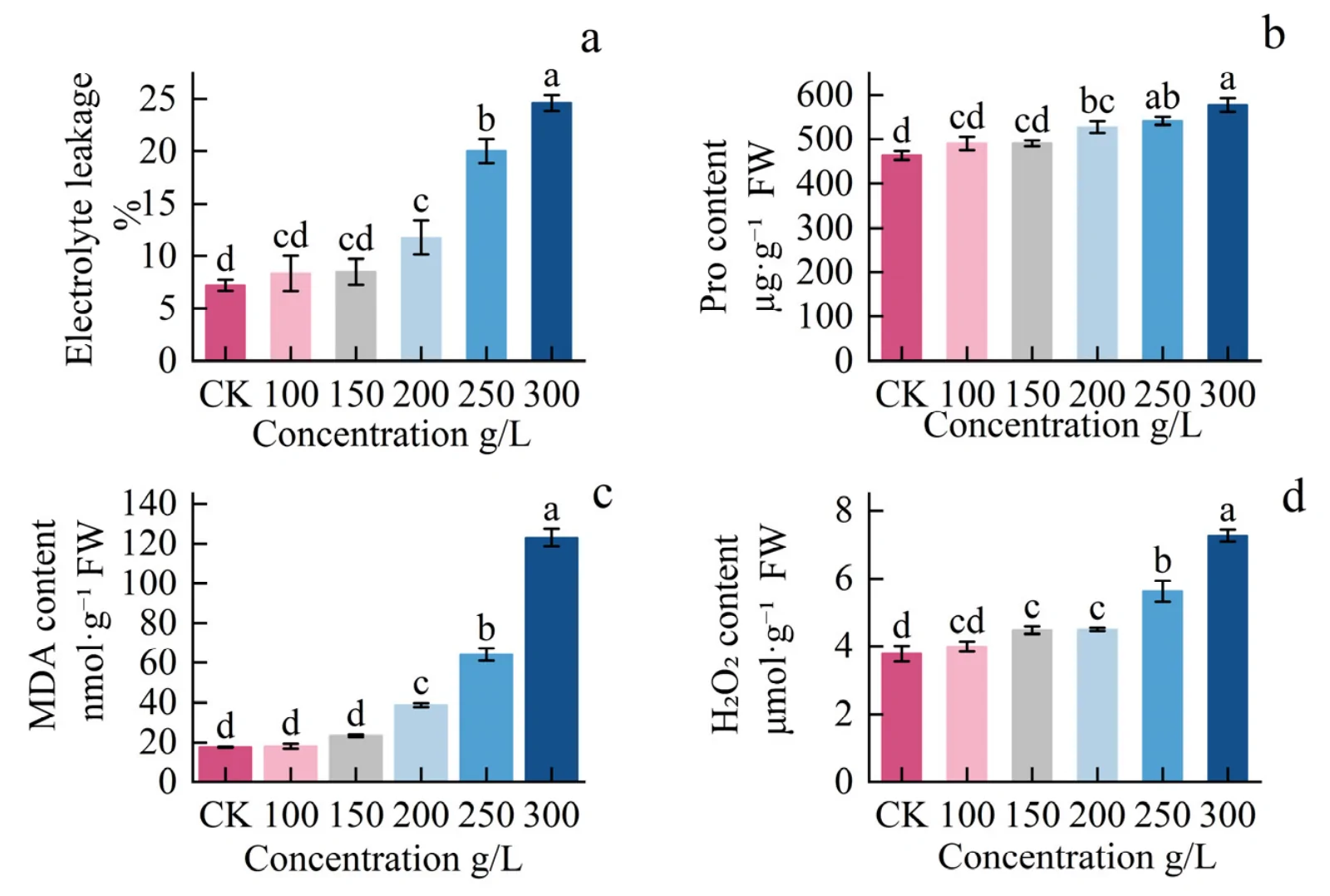
Effects of *Artemisia annua* aqueous extract on electrolyte leakage and the contents of proline, malondialdehyde, and hydrogen peroxide in *Solanum rostratum* leaves. Note: (**a**) electrolyte leakage; (**b**) proline content; (**c**) malondialdehyde content; (**d**) hydrogen peroxide content. Different lowercase letters indicate significant differences among treatments at *p* < 0.05. Data are presented as means ± SE (*n* = 3 independent seedlings per treatment).

Electrolyte leakage and proline content in *S. rostratum* leaves generally increased with increasing concentrations of the *A. annua* aqueous extract (Figure 5a,b). Electrolyte leakage was 7.22% in the CK treatment (Figure 5a) and increased slightly to 8.34% and 8.50% under the 100 and 150 g L^−1^ treatments, respectively; however, neither value differed significantly from the CK value (*p* > 0.05). At extract concentrations of 200, 250, and 300 g L^−1^, electrolyte leakage was significantly higher than that in the CK, with values of 11.17%, 20.02%, and 24.62%, respectively (*p* < 0.05).

Proline content increased progressively with increasing extract concentration (Figure 5b). Proline content was 462.98 μg·g^−1^ in the CK treatment and increased to 489.91 and 490.21 μg·g^−1^ under the 100 and 150 g L^−1^ treatments, respectively. Although these values were higher than the CK value, the differences were not significant (*p* > 0.05). Under the 200 and 250 g L^−1^ treatments, proline content increased significantly to 527.53 and 541.68 μg·g^−1^, respectively (*p* < 0.05). The highest proline content, 577.44 μg·g^−1^, was recorded at 300 g L^−1^ and was significantly higher than the CK value (*p* < 0.05).

Malondialdehyde (MDA) also increased with increasing extract concentration (Figure 5c). MDA content was 17.77 nmol·g^−1^ in the CK treatment and increased to 18.22 and 23.33 nmol·g^−1^ under the 100 and 150 g L^−1^ treatments, respectively; however, neither value differed significantly from the CK value (*p* > 0.05). At concentrations of 200, 250, and 300 g L^−1^, MDA content was significantly higher than that in the CK, with values of 38.80, 63.98, and 123.03 nmol·g^−1^, respectively (*p* < 0.05).

Hydrogen peroxide (H_2_O_2_) content increased continuously with increasing extract concentration (Figure 5d). The highest H_2_O_2_ content was recorded under the 300 g L^−1^ treatment, reaching 7.27 μmol·g^−1^, which was 92.58% higher than the CK value.

**Figure 6:**
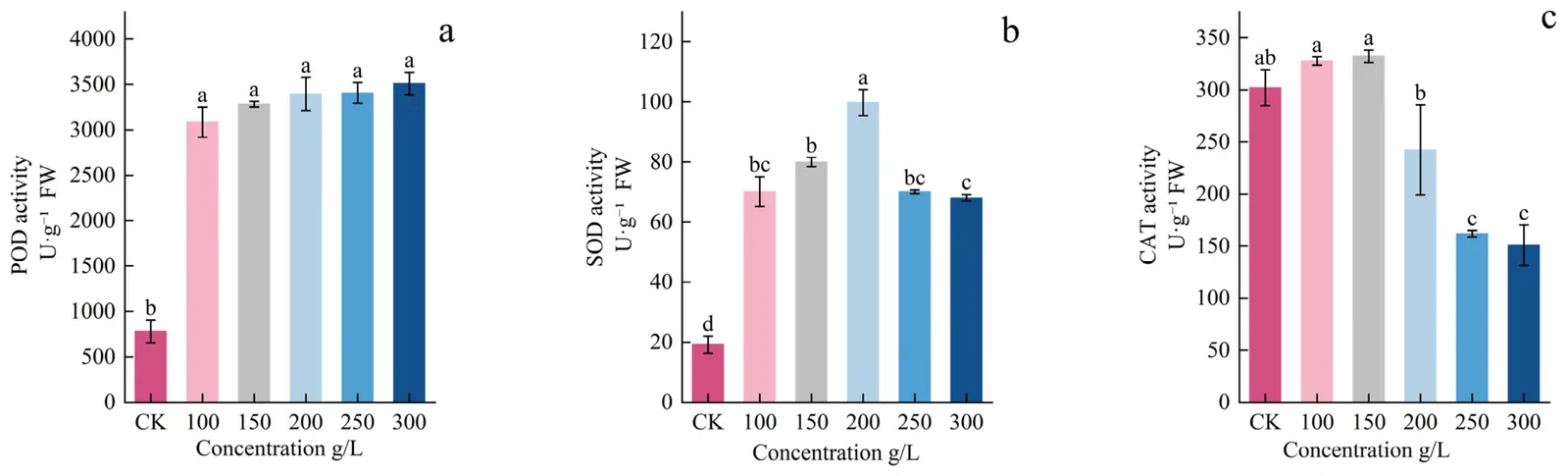
Effects of *Artemisia annua* aqueous extract on antioxidant enzyme activities in *Solanum rostratum* leaves. Note: (**a**) peroxidase (POD) activity; (**b**) superoxide dismutase (SOD) activity; (**c**) catalase (CAT) activity. Different lowercase letters indicate significant differences among treatments at *p* < 0.05. Data are presented as means ± SE (*n* = 3 independent seedlings per treatment).

The activities of antioxidant enzymes in *S. rostratum* leaves varied with the concentration of the *A. annua* aqueous extract (Figure 6). Peroxidase (POD) activity was significantly higher under all extract treatments than under the CK treatment (*p* < 0.05; Figure 6a), whereas no significant differences were detected among the extract treatments (*p* > 0.05).

Superoxide dismutase (SOD) activity initially increased and subsequently decreased with increasing extract concentration but remained higher than the CK value (Figure 6b). The highest SOD activity, 99.78 U·g^−1^, was recorded under the 200 g L^−1^ treatment. Although SOD activity declined at 250 and 300 g L^−1^, it remained significantly higher than the CK value (*p* < 0.05).

Catalase (CAT) activity was numerically higher under the 100 and 150 g L^−1^ treatments than under the CK treatment, although the differences were not significant (*p* > 0.05; Figure 6c). In contrast, CAT activity was significantly lower under the 200–300 g L^−1^ treatments than under the CK treatment (*p* < 0.05). Specifically, CAT activity decreased by 46.38% and 50.10% under the 250 and 300 g L^−1^ treatments, respectively, relative to the CK treatment.

## 3. Discussion

### 3.1. Chemical Characteristics of the Artemisia annua Aqueous Extract

LC–MS/MS analysis yielded a chemically diverse set of putative annotations in the *A. annua* aqueous extract. The annotation entries spanned ten superclasses, with phenylpropanoids, alkaloids, and terpenoids being the most represented by number of entries. Several entries assigned to phenylpropanoids, including heraclenol, β-D-glucosylisoeugenol, 4-[2-hydroxy-3-(4-hydroxyphenyl)propyl]phenol, and salvianolic acid C, were among those with the ten highest mean LC–MS/MS peak-area responses. The dataset also included a putative annotation for artemisinin, although this entry was not among the ten highest-ranked entries. Collectively, these findings are consistent with the chemical complexity of the tested crude aqueous extract rather than a chemically defined preparation containing a single constituent.

Several of the putatively annotated compounds or compound classes have previously been associated with phytotoxic or allelopathic activity. For example, artemisinin has been reported to reduce photosynthetic performance, increase electrolyte leakage, and alter antioxidant enzyme activities in recipient plants [16]. The annotation dataset also included entries assigned to coumarins, flavonoids, phenolic compounds, and terpenoid-related metabolites. Compounds within these classes have been associated with the inhibition of seed germination and plant growth in previous studies [11,12]. These literature reports provide biological plausibility for the phytotoxic responses observed in the present study. However, they do not establish that the corresponding compounds were responsible for the responses of *S. rostratum*. The effects of the crude extract may instead reflect the combined actions of multiple constituents, together with concentration-dependent physicochemical properties of the extract.

The present LC–MS/MS results therefore do not demonstrate that any individual metabolite caused the observed responses. Because chemical profiling was performed only on the 300 g L^−1^ extract, the data do not establish the composition or relative abundance of annotated metabolites at the lower concentrations used in the bioassays. In addition, LC–MS/MS peak-area magnitude depends not only on metabolite concentration but also on compound-specific ionization and fragmentation efficiencies. The annotations were not confirmed using authentic standards, and compound-specific calibration curves were unavailable. Moreover, because the pH, electrical conductivity, osmotic potential, and dissolved nutrient content of the extract solutions were not independently controlled, their potential contributions to the observed responses cannot be excluded. Confirmation using authentic standards, quantitative chemical analysis, physicochemical controls, and compound-specific dose–response bioassays would therefore be required before the observed phytotoxic effects could be attributed to particular *A. annua* metabolites.

### 3.2. Inhibitory Effects of Artemisia annua Aqueous Extract on Solanum rostratum Seed Germination

Seed production and dispersal play critical roles in the invasion and spread of *S. rostratum*. Its prolific seed production is considered an important factor contributing to its rapid and extensive invasion [18,19]. Seed germination therefore represents a relevant stage for limiting the establishment of *S. rostratum*.

In the present study, the germination rate, germination energy, and germination index of *S. rostratum* were all zero at *A. annua* aqueous extract concentrations ≥150 g L^−1^. Similar inhibitory effects of plant extracts on weed seed germination have been reported previously. For example, a methanolic extract of *Artemisia santolinifolia* was reported to inhibit the germination of *Sinapis arvensis* and *Lolium multiflorum* [17]. Similarly, an aqueous leaf extract of *Anacardium occidentale* at 50 mg·mL^−1^ completely inhibited the germination of *Lactuca sativa* seeds [20]. Although the donor species, extraction procedures, treatment concentrations, and recipient species differed among these studies, their results collectively show that plant-derived extracts can restrict seed germination and early development. The present findings show that S. rostratum exhibits a similar response to *A. annua* aqueous extract.

At 50 and 100 g L^−1^, some *S. rostratum* seeds germinated, but the germination rate, germination energy, germination index, and recorded mean radicle and plumule lengths were lower than the corresponding CK values. These concurrent reductions indicate that the *A. annua* aqueous extract suppressed seed germination and overall early development.

The inhibition of germination and early development may be associated with disruption of the physiological processes required for embryo growth. Previous studies have shown that plant-derived allelochemicals can inhibit hydrolytic enzymes involved in the mobilization of stored reserves, potentially restricting the energy and metabolic substrates available for embryo development [21]. Allelochemicals have also been reported to promote excessive accumulation of reactive oxygen species (ROS), impair membrane integrity, and cause cellular injury in germinating seeds [22]. These processes could limit the energy supply and cellular function required for embryo growth, thereby delaying or preventing radicle emergence. However, hydrolytic enzyme activities, ROS accumulation, and membrane integrity in *S. rostratum* seeds were not measured in the present study.

The present results provide laboratory evidence that *A. annua* aqueous extract inhibited *S. rostratum* seed germination and overall early development. Whether these effects persist in soil and influence seedling establishment or soil seed-bank dynamics remains to be evaluated under field conditions.

### 3.3. Contrasting Morphological and Physiological Responses of Solanum rostratum Seedlings

Because buried seeds of *S. rostratum* can remain viable and emerge when environmental conditions become favorable, seedling responses are relevant to the subsequent establishment of this species [19]. In the present study, several aboveground morphological traits, including the net increases in plant height and basal diameter, leaf area, leaf length, leaf width, and leaf thickness, increased under the 100–150 g L^−1^ treatments. Some root morphological traits also showed numerical increases at these concentrations, although most differences from the CK were not statistically significant. At concentrations ≥200 g L^−1^, aboveground and root morphological traits generally decreased, indicating a shift toward morphological inhibition. Similar concentration-dependent biphasic patterns have been reported for other plant extracts. For example, an aqueous extract of *Artemisia frigida* increased shoot growth in lettuce (*Lactuca sativa*) seedlings at a relatively low concentration but inhibited it at a higher concentration [23]. Similarly, lower concentrations of *Solidago canadensis* aqueous extract increased several morphological traits in maize (*Zea mays*) seedlings, whereas higher concentrations suppressed these traits [24].

The increases in selected morphological traits at 100–150 g L^−1^ did not coincide with improved gas-exchange performance. Net photosynthetic rate, stomatal conductance, and transpiration rate were significantly lower than the corresponding CK values at both concentrations. The concurrent increase in selected morphological traits and decline in gas-exchange performance suggests that these morphological changes may represent plastic adjustment under physiological stress rather than greater whole-plant growth. Because whole-plant biomass was not measured, the present data cannot determine whether the higher morphological values reflected greater biomass accumulation.

Several non-exclusive processes could account for these contrasting morphological and physiological responses. Because the treatment consisted of a crude aqueous extract, soluble nutrients and bioactive organic constituents may have contributed to the increases in selected morphological traits, whereas other phytotoxic constituents may have contributed to the concurrent physiological stress [25,26]. The response may also be consistent with a hormetic-type pattern, in which relatively low exposure increases selected traits, whereas higher exposure becomes inhibitory [27,28]. Alternatively, the increases in plant height and leaf dimensions may reflect stress-related adjustments in organ expansion and biomass allocation—components of morphological plasticity—rather than greater whole-plant biomass [29,30]. These explanations are not mutually exclusive and may have contributed concurrently to the observed responses.

At concentrations ≥200 g L^−1^, reductions in both aboveground and root morphological traits became increasingly evident, with the lowest values for several traits observed at 300 g L^−1^. Root length, surface area, volume, and branching were substantially reduced under the higher-concentration treatments. Because the extract was applied to the root zone, direct root exposure may have contributed to these responses. Previous studies have shown that plant-derived compounds can affect root-meristem activity, cell division, cell elongation, and ROS-related processes [31,32]. Because root systems regulate water and mineral nutrient acquisition [33], reduced root development at higher extract concentrations may have contributed to the reductions in aboveground morphological traits, although this relationship was not directly assessed.

The seedling experiment began at 100 g L^−1^ and did not include the 50 g L^−1^ concentration used in the germination assay. The response of established seedlings at 50 g L^−1^ and the lower concentration boundary of the observed morphological increases therefore remain unknown. Moreover, because the germination and seedling assays differed in both concentration range and experimental system, their results do not support a direct comparison of sensitivity between the two developmental stages. Future studies using a common, more finely resolved concentration series and measurements of whole-plant biomass would help distinguish increased biomass accumulation from morphological plasticity or transient stress responses.

### 3.4. Physiological Responses of Solanum rostratum Seedlings to Artemisia annua Aqueous Extract

Photosynthesis provides the physiological basis for biomass production and dry-matter accumulation in plants [34]. In the present study, net photosynthetic rate (*P_n_*), stomatal conductance (*g_s_*), and transpiration rate (*T_r_*) of *S. rostratum* seedlings decreased progressively with increasing extract concentration. By contrast, intercellular CO_2_ concentration (*C_i_*) initially decreased slightly and then increased significantly at extract concentrations ≥200 g L^−1^. The significant decrease in *g_s_* indicates that stomatal limitation may have contributed to the reduction in photosynthesis. However, at extract concentrations ≥200 g L^−1^, *P_n_* continued to decline while *C_i_* increased, suggesting that limitations to mesophyll CO_2_ assimilation may also have contributed to the reduction in *P_n_* [35]. A comparable response has been reported in *Vatica mangachapoi* seedlings exposed to *Casuarina equisetifolia* leachates, which reduced carboxylation efficiency and increased the CO_2_ compensation point [36]. Given the variation in chamber temperature, relative humidity, and reference CO_2_ concentration during measurements, the gas-exchange results are interpreted as relative treatment responses within the semi-open greenhouse environment.

Carotenoids function as nonenzymatic antioxidants and photoprotective pigments, and their synthesis or accumulation can help plants alleviate stress-induced damage [37,38]. In the present study, chlorophyll a, chlorophyll b, and total chlorophyll contents initially increased, reached their highest values at 150 g L^−1^, and then declined with further increases in extract concentration. In contrast, carotenoid content increased across the tested concentration gradient. Previous studies have reported that plant-derived allelochemicals may alter chlorophyll biosynthesis or degradation and affect chloroplast structure, thylakoid membranes, electron transport, and photosystem function [39,40,41]. The biphasic chlorophyll response observed in the present study may reflect adjustments in pigment metabolism or chloroplast function, whereas the progressive increase in carotenoid content may represent photoprotective or antioxidant adjustment.

Under stressful conditions, the balance between ROS production and scavenging is frequently disrupted [42]. In the present study, increasing extract concentration was accompanied by increases in H_2_O_2_, MDA, electrolyte leakage, and proline, together with concentration-dependent changes in POD, SOD, and CAT activities. The increases in H_2_O_2_ and MDA, together with greater electrolyte leakage at higher concentrations, are consistent with increasing oxidative stress and elevated indicators of membrane damage. Similar changes in lipid-peroxidation indicators, membrane permeability, and antioxidant enzyme activities have been reported in plants exposed to plant-derived phytotoxic substances [43,44]. The higher POD activity and the numerical increases in SOD and CAT activities at relatively lower concentrations may reflect antioxidant adjustment. By contrast, the decline in CAT activity at concentrations ≥200 g L^−1^ coincided with increasing H_2_O_2_ content, suggesting a reduced contribution of CAT-mediated H_2_O_2_ scavenging at higher extract concentrations. The increase in proline may reflect osmotic adjustment as part of the broader stress response. The concurrent changes in oxidative-stress indicators, membrane-damage indicators, proline content, and antioxidant enzyme activities are consistent with an integrated physiological stress response to increasing extract concentration.

Across the tested concentration gradient, gas-exchange suppression was evident at all extract concentrations, whereas oxidative-stress and membrane-damage indicators became more pronounced at higher concentrations. This pattern indicates that the physiological stress response broadened as extract concentration increased.

### 3.5. Ecological Considerations and Future Perspectives

Because the treatments consisted of crude aqueous extracts, the observed responses may reflect both the activities of extract constituents and concentration-dependent physicochemical properties of the solutions. The pH, electrical conductivity, and osmotic potential of the different extract concentrations were not measured, and repeated application may also have promoted the accumulation of dissolved constituents in the root zone. The effects observed at higher concentrations are best interpreted as responses to the whole aqueous extract under the present experimental conditions rather than exclusively to allelochemical activity. Physicochemical measurements and corresponding controls would be needed to distinguish metabolite-associated phytotoxicity from nonspecific concentration effects.

The present study provides laboratory evidence that *S. rostratum* responds to *A. annua* aqueous extract in a concentration-dependent manner, but it does not establish target selectivity or field efficacy. Selected morphological traits increased at 100–150 g L^−1^, whereas morphological inhibition became increasingly evident at concentrations ≥200 g L^−1^. This pattern indicates that field exposure below the inhibitory range might not suppress the target species. Moreover, responses to plant-derived constituents may differ among recipient species, potentially reflecting differences in detoxification and metabolism as well as intrinsic stress tolerance [45,46]. Comparative dose–response experiments involving *S. rostratum*, co-occurring native species, and locally important crops and forage plants are needed to evaluate target efficacy and non-target safety.

The plug-tray experiment represented a simplified root-zone exposure system. Under field conditions, extract constituents may undergo adsorption, degradation, leaching, and microbial transformation, and their exposure patterns may differ substantially from the repeated applications used here. The concentrations associated with morphological and physiological responses in this study should be regarded as experimental treatment ranges rather than field application thresholds.

## 4. Materials and Methods

### 4.1. Plant Materials

The aboveground parts of flowering *Artemisia annua* plants were collected in August 2025 near Inner Mongolia Agricultural University in Hohhot, China. The material was shade-dried to constant weight, ground to a powder, and used to prepare aqueous extracts for both the seed germination and seedling experiments. No voucher specimen was prepared or deposited for *A. annua*.

Seeds of *Solanum rostratum* were collected in October 2024 from mature fruits of multiple maternal plants growing in an abandoned roadside field in Tumed Left Banner, Hohhot, Inner Mongolia, China. The seeds were pooled, air-dried, and cleaned to remove poorly developed and shriveled seeds. They were subsequently stored in paper bags at 4 °C under dry, dark conditions until use.

### 4.2. Preparation of the Aqueous Extract

The powdered *Artemisia annua* material described above was used without sieving. Portions of 50, 100, 150, 200, 250, and 300 g were separately added to 1 L of distilled water and soaked for 24 h. The mixtures were then filtered through gauze to obtain *A. annua* aqueous extracts with nominal concentrations of 50, 100, 150, 200, 250, and 300 g L^−1^, calculated from the dry mass of plant material added per litre of distilled water. The extracts were stored at 4 °C and used within 24 h of preparation. Extracts at 50, 100, 150, 200, and 250 g L^−1^ were used in the seed germination assay, whereas those at 100, 150, 200, 250, and 300 g L^−1^ were used in the seedling assay.

### 4.3. LC–MS/MS Profiling of the Aqueous Extract

Three independently prepared samples of the 300 g L^−1^ *Artemisia annua* aqueous extract were subjected to widely targeted LC–MS/MS analysis. Each sample was thawed on ice and vortexed for 30 s. A 200 μL aliquot was evaporated to dryness and reconstituted in 200 μL of methanol/water (4:1, *v*/*v*) containing internal standards. After vortexing for 30 s and sonication in an ice-water bath for 10 min, the samples were centrifuged at 12,000 rpm and 4 °C for 15 min. The resulting supernatants were passed through 0.22 μm membrane filters before instrumental analysis.

Chromatographic separation was performed using a Vanquish ultrahigh-performance liquid chromatography system (Thermo Fisher Scientific, Waltham, MA, USA) equipped with a Kinetex C18 column (2.1 mm × 50 mm, 2.6 μm; Phenomenex, Torrance, CA, USA). Mobile phase A consisted of water containing 0.01% acetic acid, and mobile phase B consisted of isopropanol/acetonitrile (1:1, *v*/*v*). The column and autosampler temperatures were maintained at 25 and 4 °C, respectively. The injection volume was 1 μL, and the flow rate was 0.30 mL min^−1^. The proportion of mobile phase B was maintained at 1% from 0.00 to 0.50 min, increased linearly to 99% from 0.50 to 4.00 min, and maintained at 99% until 4.50 min. It was then returned to 1% by 4.55 min and maintained at 1% until 6.00 min for column re-equilibration. The total run time was 6.00 min.

Mass-spectrometric analysis was performed using a Stellar quadrupole linear ion-trap mass spectrometer (Thermo Fisher Scientific, Waltham, MA, USA) equipped with a heated electrospray ionization source and operated in parallel reaction monitoring mode. The spray voltages were 3.8 kV in positive-ion mode and −3.4 kV in negative-ion mode. The sheath gas, auxiliary gas, and sweep gas were set to 50, 15, and 1 arbitrary units, respectively. The ion-transfer tube and vaporizer temperatures were 320 and 400 °C, respectively.

Peak extraction and automated data processing were performed using Biobud v2.0.7 software provided by Hangzhou Lianchuan Biotechnology Co., Ltd. (Hangzhou, China). Putative metabolite annotations were assigned by comparison with the company’s in-house database (PDB v6.0) using precursor-ion, product-ion, retention-time, co-elution, and MS/MS information. In parallel reaction monitoring mode, a product ion with suitable stability and specificity was selected for relative peak-area measurement. The annotations were not confirmed by analyzing authentic reference standards alongside the samples.

Six internal-standard records were excluded before data summarization. The remaining annotation entries were assigned to metabolite superclasses according to the database classification, and the number and proportion of entries in each superclass were calculated. For each annotation entry, the mean peak area was calculated across the three independently prepared samples. The entries were ranked in descending order according to mean peak-area response, and the ten highest-ranked entries were reported. Because compound-specific calibration curves were unavailable and ionization efficiencies can differ among compounds, the peak areas were interpreted as relative instrumental responses rather than metabolite concentrations or measures of relative abundance.

### 4.4. Seed Germination Experiment

The seed germination experiment was conducted using the Petri-dish filter-paper method. Fully developed, uniformly sized *Solanum rostratum* seeds were selected and surface-sterilized in a 0.5% potassium permanganate solution for 15 min. The seeds were then rinsed repeatedly with distilled water until no visible residue remained on their surfaces and soaked in distilled water for 24 h before the experiment. The seeds were placed in Petri dishes lined with filter paper. Each dish initially received 10 mL of *Artemisia annua* aqueous extract at 50, 100, 150, 200, or 250 g L^−1^. Thereafter, 5 mL of the corresponding extract was added daily to maintain filter-paper moisture. Control dishes received the same volumes of distilled water. Each treatment comprised three independent Petri dishes, with 40 seeds per dish.

Previous research showed that the optimum temperature for *S. rostratum* seed germination ranged from 25 to 35 °C and that germination did not differ significantly between a 14 h photoperiod and continuous darkness [47]. To approximate the light-limited conditions experienced by seeds buried in soil, the dishes were incubated at 28 °C under continuous darkness for 20 d. They were removed briefly from the incubator every 24 h to record the number of germinated seeds and returned immediately after observation. A seed was considered germinated when its radicle protruded 1–2 mm through the seed coat. The experiment was terminated on day 20.

### 4.5. Seedling Growth Experiment

The seedling growth experiment was conducted in a semi-open greenhouse under natural light and ambient temperatures throughout the cultivation period. Field soil collected from Inner Mongolia Agricultural University in Hohhot, China, was used as the growth substrate. The field-moist soil was passed through a 3 mm sieve without prior air-drying. The soil contained 25.66 mg kg^−1^ alkali-hydrolyzable N, 0.083 g kg^−1^ total N, 0.727 mg kg^−1^ available P, 0.53 g kg^−1^ total P, 105.72 mg kg^−1^ available K, 2.03 g kg^−1^ total K, and 9.70 g kg^−1^ soil organic carbon.

Fully developed, uniformly sized *S. rostratum* seeds were selected, surface-sterilized using the procedure described in Section 4.4, and sown individually in soil-filled planting cells. Each planting cell had an upper diameter of 6 cm, a depth of 5.3 cm, and a volume of 110 mL. One seedling was grown in each cell, and 40 independent cells were assigned to each treatment. The cells did not share soil, irrigation solution, or drainage water, and each cell received its assigned treatment separately. All cells were randomly positioned in the greenhouse at the beginning of the experiment and rearranged periodically during cultivation to minimize potential spatial effects. Each independently treated cell containing one seedling was regarded as an experimental unit.

Before treatment initiation, the seedlings were watered every 3 d. When the seedlings had developed two true leaves, 10 seedlings were randomly selected from the 40 independent experimental units assigned to each treatment, and their initial plant height, basal diameter, leaf number, and leaf thickness were measured. The seedlings were then treated with *A. annua* aqueous extract at concentrations of 100, 150, 200, 250, or 300 g L^−1^ by root irrigation. Each planting cell received 30 mL of the corresponding extract every 3 d for 6 weeks, resulting in 14 applications. No additional water was supplied during the treatment period. Control cells received 30 mL of distilled water at the same application intervals.

After 6 weeks of treatment, plant height, basal diameter, leaf number, and leaf thickness were remeasured in the same 10 seedlings. These 10 independently treated seedlings served as the biological replicates for these measurements. Leaf area was determined in five independent seedlings per treatment. Additional measurements included root morphological traits and gas-exchange parameters, namely the net photosynthetic rate (*P_n_*), transpiration rate (*T_r_*), stomatal conductance (*g_s_*), and intercellular CO_2_ concentration (*C_i_*). Photosynthetic pigments included chlorophyll a, chlorophyll b, total chlorophyll, and carotenoids. Stress-related variables included electrolyte leakage, malondialdehyde (MDA), peroxidase (POD), superoxide dismutase (SOD), catalase (CAT), hydrogen peroxide (H_2_O_2_), and proline.

### 4.6. Measurements and Calculations

#### 4.6.1. Seed Germination Parameters

Germination parameters and indices were calculated separately for each independent Petri dish. Germination rate (GR), germination energy (GE), and germination index (GI) were calculated as follows:

GR (%) = (cumulative number of seeds germinated at the end of the experiment/total number of seeds) × 100

GE (%) = (cumulative number of seeds germinated up to and including the peak germination day/total number of seeds) × 100

GI = Σ(G_t_/D_t_)

For each Petri dish, the peak germination day was defined as the day on which the greatest number of newly germinated seeds was recorded. In the GI calculation, G_t_ is the number of seeds that newly germinated on day t, and D_t_ is the corresponding day number.

The synthetic allelopathic effect index (SE) was calculated as follows:

SE = (RI_GR + RI_GE + RI_GI + RI_RL + RI_PL)/5

where RI_GR, RI_GE, RI_GI, RI_RL, and RI_PL represent the response indices for germination rate, germination energy, germination index, radicle length, and plumule length, respectively. When mean radicle and plumule lengths were calculated, non-germinated seeds were assigned length values of zero. These mean values were subsequently used to calculate RI_RL and RI_PL. The response index (RI) was calculated as follows:

RI = 1 − C/T, when T ≥ C

RI = T/C − 1, when T < C

where C and T represent the corresponding values for the control and extract treatments, respectively. Positive RI or SE values indicate stimulation, whereas negative values indicate inhibition.

#### 4.6.2. Seedling Morphological and Physiological Measurements

Plant height was measured using a ruler, whereas basal diameter and leaf thickness were measured using a Vernier caliper (Wynn’s Holding Group Co., Ltd., Foshan, China). Leaf number was counted, and leaf area was determined after 6 weeks of treatment. To account for differences in initial seedling size, net increments in plant height, basal diameter, leaf number, and leaf thickness were calculated. Net increment was calculated as follows:

Net increment = final value − initial value.

For root morphological measurements, seven seedlings were randomly selected from the 40 independent experimental units in each treatment and used as biological replicates. Each root system was scanned separately, and the resulting images were analyzed using WinRHIZO Pro 2012b software (Regent Instruments Inc., Quebec City, QC, Canada). The measured traits included total root length, root surface area, root volume, number of root tips, number of root forks, and the length of roots with diameters ≤0.5 mm.

Leaf gas-exchange parameters were measured between 09:00 and 11:00 using a GFS-3000 portable gas-exchange system (Heinz Walz GmbH, Effeltrich, Germany) in the semi-open greenhouse. Five seedlings were randomly selected from the 40 independent experimental units in each treatment. The second fully expanded leaf from the top of each seedling was measured, with one leaf representing one biological replicate (*n* = 5 per treatment). The integrated red–blue light source provided a photosynthetic photon flux density of 1000 μmol m^−2^ s^−1^. The nominal chamber temperature and reference CO_2_ concentration were set to 25 °C and 400 μmol mol^−1^, respectively. During measurement, the recorded chamber temperature ranged from 30.55 to 31.93 °C, relative humidity from 34.62% to 45.41%, and reference CO_2_ concentration from 475.55 to 593.92 μmol mol^−1^. The chamber flow rate was approximately 750 μmol s^−1^. After the readings had stabilized, three readings were recorded from each leaf at intervals of approximately 30 s and averaged to obtain one value for each biological replicate. The recorded parameters were net photosynthetic rate (*P_n_*), transpiration rate (*T_r_*), stomatal conductance (*g_s_*), and intercellular CO_2_ concentration (*C_i_*).

For photosynthetic pigment, electrolyte leakage, and biochemical measurements, functional leaves were collected from three randomly selected seedlings per treatment. Samples from individual seedlings were processed separately, and each seedling represented one biological replicate (*n* = 3 per treatment).

Photosynthetic pigment contents were determined according to Qiu et al., with minor modifications [48]. Approximately 0.05 g of fresh leaf tissue from each seedling was cut into strips approximately 1 mm wide and placed in a stoppered 10 mL tube containing 2 mL of dimethyl sulfoxide (DMSO). The samples were extracted at 65 °C for 1.5 h in darkness. After cooling to room temperature, 8 mL of 80% (*v*/*v*) acetone was added. The tubes were immediately sealed and vortexed for at least 30 s. A mixture of 2 mL of DMSO and 8 mL of 80% acetone was used as the spectrophotometric blank. Absorbance was measured at 470, 646.6, and 663.6 nm. Three technical readings were obtained from each extract and averaged to produce one value for each biological replicate.

The concentrations of chlorophyll a and chlorophyll b in the extract were calculated as follows:

C_a_ (mg L^−1^) = 12.27A_663_ − 2.52A_646_

C_b_ (mg L^−1^) = 20.10A_646_ − 4.92A_663_

Carotenoid concentration was calculated as follows:

Cx + c (mg L^−1^) = (1000A_470_ − 3.27C_a_ − 104C_b_)/229

Pigment contents were expressed on a fresh-weight basis using the following equation:

Pigment content (mg g^−1^ FW) = C × V/m

where C is the pigment concentration in the extract (mg L^−1^), V is the final extract volume (0.010 L), and m is the actual fresh mass of the leaf sample (g). Total chlorophyll content was calculated as the sum of chlorophyll a and chlorophyll b contents.

Electrolyte leakage was determined using the conductivity method described by Gu and Lu, with minor modifications [49]. Approximately 0.1 g of fresh leaf tissue from each seedling was rinsed with deionized water and placed in a test tube containing 10 mL of deionized water. The samples were incubated at room temperature for 2 h and handled carefully to minimize tissue damage. After the tubes had been gently inverted, the initial electrical conductivity (C_1_) was measured. The sealed tubes were then heated in a boiling-water bath for 10 min to release the electrolytes. After cooling to room temperature, the final electrical conductivity (C_2_) was measured. Electrolyte leakage was calculated as follows:

Electrolyte leakage (%) = C_1_/C_2_ × 100

Malondialdehyde (MDA), hydrogen peroxide (H_2_O_2_), and proline contents and the activities of peroxidase (POD), superoxide dismutase (SOD), and catalase (CAT) were determined using commercial assay kits supplied by Beijing Solarbio Science & Technology Co., Ltd. (Beijing, China). Approximately 0.1 g of fresh leaf tissue from each seedling was extracted with 1.0 mL of the extraction solution supplied with the corresponding kit. All assays were performed according to the manufacturer’s instructions.

For MDA determination, absorbance was measured at 532 and 600 nm, with the value at 600 nm used to correct for nonspecific absorbance. H_2_O_2_ content was determined from absorbance at 415 nm, whereas proline content was determined from absorbance at 520 nm. POD activity was determined from the change in absorbance at 470 nm, SOD activity from absorbance at 560 nm, and CAT activity from the change in absorbance at 240 nm. The actual fresh mass of each sample was used in the calculations. MDA, H_2_O_2_, and proline contents and POD, SOD, and CAT activities were expressed on a fresh-weight basis and calculated according to the manufacturer’s instructions.

### 4.7. Statistical Analysis

Experimental data were organized using Microsoft Excel 2010. Statistical analyses were performed using SPSS version 26.0 and R version 4.4 (R Foundation for Statistical Computing, Vienna, Austria). Data are presented as observed means ± standard error (SE). The seed-germination assay comprised three independent Petri dishes per treatment, with 40 seeds per dish. In the seedling experiment, the biological sample sizes per treatment were 10 seedlings for plant height, basal diameter, leaf number, and leaf thickness; five seedlings for leaf area, leaf length, leaf width, and gas-exchange measurements; seven seedlings for root morphological measurements; and three seedlings for photosynthetic pigment and physiological measurements. Repeated instrumental readings from the same biological sample were averaged before statistical analysis and were not treated as independent biological replicates.

Germination rate (GR) and germination energy (GE) were analyzed as grouped binomial responses using the corresponding numbers of germinated and non-germinated seeds in each Petri dish, with 40 seeds representing the number of trials per dish. Because no germination was observed at extract concentrations ≥150 g L^−1^, resulting in complete separation, mean bias-reduced binomial generalized linear models with a logit link (Firth-type correction) were fitted using the brglm2 package (version 1.0.1) in R [50,51]. Extract concentration was included as a fixed categorical factor. Overall treatment effects were evaluated using Wald χ^2^ tests. Pairwise contrasts were obtained using the emmeans package (version 2.0.4), with the Holm procedure applied to adjust for multiple comparisons. For graphical presentation, GR and GE were presented as the observed mean percentages ± SE across the three independent Petri dishes, whereas the significance letters were based on the Holm-adjusted model comparisons. Germination index, radicle length, plumule length, and the synthetic allelopathic effect index were analyzed using the independent Petri dishes as replicates. To account for the zero-variance groups and small sample sizes, overall treatment effects were evaluated by permutation tests of the Kruskal–Wallis statistic in R. Tied values were assigned average ranks. All 15 pairwise comparisons among the six treatments were adjusted using the maximum absolute difference in mean ranks across comparisons in each permutation. For each variable, 99,999 permutations were performed with three Petri dishes retained per treatment; Monte Carlo *p* values were calculated with a plus-one correction and fixed random seeds.

Seedling morphological and physiological response variables were analyzed by one-way analysis of variance in IBM SPSS Statistics version 26.0. When the overall treatment effect was significant, Duncan’s multiple range test was used for pairwise comparisons. Differences were considered statistically significant at *p* < 0.05.

For visualization, selected root morphological variables were independently transformed using min–max normalization:

X_norm_ = (X − X_min_)/(X_max_ − X_min_)

where X_min_ and X_max_ were the minimum and maximum individual observations, respectively, for each variable across all treatments. Treatment means were normalized using the same equation, and the corresponding SE values were divided by X_max_ − X_min_. All descriptions in the main text and all statistical significance tests were based on the original measured data. Figures and tables were prepared using Origin 2021.

## 5. Conclusions

*Artemisia annua* aqueous extract suppressed *Solanum rostratum* seed germination at all tested concentrations, with no germination observed at concentrations ≥150 g L^−1^. Seedling responses varied with extract concentration. The net increases in plant height, basal diameter, and leaf number, together with leaf area, were higher at 100–150 g L^−1^, while leaf thickness, length, and width reached their highest values at 150 g L^−1^. These morphological responses occurred despite concurrent reductions in gas-exchange performance. At concentrations ≥200 g L^−1^, morphological inhibition became increasingly evident. Under the higher-concentration treatments, morphological inhibition coincided with reduced photosynthetic performance, increased H_2_O_2_ and MDA contents, greater electrolyte leakage, higher POD and SOD activities, and lower CAT activity. LC–MS/MS analysis generated putative metabolite annotations spanning multiple superclasses, among which phenylpropanoids, alkaloids, and terpenoids contained the largest numbers of annotation entries. Together, these findings provide integrated laboratory evidence that *A. annua* aqueous extract affects both seed germination and established seedlings of *S. rostratum* and identify physiological responses associated with seedling inhibition at higher concentrations. The results provide a basis for identifying active constituents and further evaluating the phytotoxic potential of *A. annua*-derived materials.

Because the present experiments evaluated a crude aqueous extract under controlled conditions, the observed responses cannot yet be attributed to specific metabolites or extrapolated directly to field efficacy and ecological safety. Further studies should characterize the physicochemical properties of the extract, confirm and quantify candidate compounds using authentic standards, refine the concentration–response relationship, and evaluate non-target effects, environmental fate, and efficacy under field-relevant conditions.

## Figures and Tables

**Table 1:** Distribution of putatively annotated metabolite entries among ten superclasses in the aqueous extract of *Artemisia annua*.

Metabolite Superclass	Number of Annotation Entries	Percentage (%)
Phenylpropanoids	2370	23.73
Alkaloids	2334	23.37
Terpenoids	2259	22.62
Lipids	1084	10.85
Amino acids and derivatives	674	6.75
Polyketides	510	5.11
Others	438	4.39
Carbohydrates	128	1.28
Organic acids and derivatives	120	1.20
Steroids and steroid derivatives	70	0.70

Note: The values represent putatively annotated LC–MS/MS entries rather than unequivocally identified compounds. Six internal-standard records were excluded. Percentages were calculated from the number of annotation entries assigned to each superclass and do not represent the relative concentrations or peak-area proportions of these metabolite classes in the aqueous extract.

**Table 2:** Putatively annotated metabolite entries with the ten highest mean LC–MS/MS peak-area responses in the aqueous extract of *Artemisia annua*.

Rank	Putatively Annotated Metabolite	Formula	Superclass
1	Heraclenol	C_16_H_16_O_6_	Phenylpropanoids
2	10-Methyl-2H,3H,4H,7H,10H-pyrano [3,2-c]quinoline-3,5-diol	C_13_H_15_NO_3_	Polyketides
3	β-D-Glucosylisoeugenol	C_16_H_22_O_7_	Phenylpropanoids
4	4-Penten-2-ol	C_5_H_10_O	Lipids
5	D-Phenylalanine	C_9_H_11_NO_2_	Amino acids and derivatives
6	(1R,3S,5S,6S)-6-Hydroxy-1,3-dimethoxy-2-oxaspiro [4.5]dec-7-ene-8-carbaldehyde	C_12_H_18_O_5_	Alkaloids
7	Dimethyl succinate	C_6_H_10_O_4_	Lipids
8	4-[2-Hydroxy-3-(4-hydroxyphenyl)propyl]phenol	C_15_H_16_O_3_	Phenylpropanoids
9	Salvianolic acid C	C_26_H_20_O_10_	Phenylpropanoids
10	Betaine	C_5_H_11_NO_2_	Amino acids and derivatives

**Table 3:** Effects of *Artemisia annua* aqueous extract on leaf thickness, length, and width in *Solanum rostratum* seedlings.

Treatment (g L^−1^)	Net Increase in Leaf Thickness (mm)	Leaf Length (cm)	Leaf Width (cm)
CK	0.05 ± 0.01 bc	2.03 ± 0.19 b	1.62 ± 0.13 b
100	0.08 ± 0.02 ab	2.16 ± 0.13 b	1.57 ± 0.07 b
150	0.10 ± 0.01 a	2.97 ± 0.20 a	2.21 ± 0.10 a
200	0.05 ± 0.01 bc	1.55 ± 0.07 c	1.16 ± 0.06 c
250	0.05 ± 0.01 bc	1.18 ± 0.09 cd	0.93 ± 0.04 d
300	0.03 ± 0.01 c	0.83 ± 0.06 d	0.68 ± 0.04 e

Note: Different lowercase letters indicate significant differences among treatments at *p* < 0.05. Data are presented as means ± SE (*n* = 10 independent seedlings per treatment for the net increase in leaf thickness; *n* = 5 independent seedlings per treatment for leaf length and leaf width).

## Data Availability

The data supporting the findings of this study are included in the article. Further inquiries can be directed to the corresponding author.
